# The impact of reference gene selection in quantification of gene expression levels in guinea pig cervical tissues and cells

**DOI:** 10.1186/1756-0500-6-34

**Published:** 2013-01-30

**Authors:** Annika Lindqvist, Dustin Manders, R Ann Word

**Affiliations:** 1 Department of Obstetrics and Gynecology, University of Texas Southwestern Medical Center, 5323 Harry Hines Blvd F2.302, Dallas, TX, 75390, USA

**Keywords:** Cervix, Guinea pig, qPCR, Reference genes, Parturition, Progesterone, Estradiol

## Abstract

**Background:**

Accurate measurements of mRNA expression levels in tissues or cells are crucially dependent on the use of relevant reference genes for normalization of data. In this study we used quantitative real-time PCR and two Excel-based applets (geNorm and BestKeeper) to determine the best reference genes for quantification of target gene mRNA in a complex tissue organ such as the guinea pig cervix.

**Results:**

Gene expression studies were conducted in cervical epithelium and stroma during pregnancy and parturition and in cultures of primary cells from this tissue. Among 15 reference gene candidates examined, both geNorm and BestKeeper found *CLF1* and *CLTC* to be the most stable in cervical stroma and cervical epithelium, *ACTB* and *PPIB* in primary stroma cells, and *CLTC* and *PPIB* in primary epithelial cells. The order of stability among the remaining candidate genes was not in such an agreement. Commonly used reference such as *GAPDH* and *B2M* demonstrated lower stability. Determination of pairwise variation values for reference gene combinations using geNorm revealed that the geometric mean of the two most stable genes provides sufficient normalization in most cases. However, for cervical stroma tissue in which many reference gene candidates displayed low stability, inclusion of three reference genes in the geometric mean may improve accuracy of target gene expression level analyses. Using the top ranked reference genes we examined the expression levels of target gene *PTGS2* in cervical tissue and cultured cervical cells. We compared the results with *PTGS2* expression normalized to the least stable gene and found significant differences in gene expression, up to 10-fold in some samples, emphasizing the importance of appropriately selecting reference genes.

**Conclusions:**

We recommend using the geometric mean of *CFL1* and *CLTC* for normalization of qPCR studies in guinea pig cervical tissue studies, *ACTB* and *PPIB* in primary stroma cells and *CLTC* and *PPIB* in primary epithelial cells from guinea pig.

## Background

Accurate measurement of relative mRNA expression levels in tissues or cells using quantitative real-time PCR (qPCR) is crucially dependent on normalization of the data. Due to overall differences in transcriptional activity between tissues and different cell types, normalization cannot be based simply on the amounts of starting material. Since all mRNA molecules in one sample are subject to the same efficiency of RNA purification, reverse transcription and polymerase amplification, quantification of a target gene in the sample is better normalized using the ratio of the target mRNA to an endogenous mRNA reference gene within the sample. It is imperative to use reference genes with minimal variability between samples that are not influenced by the study conditions. A multitude of reference genes have been utilized for this purpose, although none are universally applicable for all tissues or cell types [1,2]. Thus, for every new experimental system, it is important to identify reference genes that fulfill these criteria. In addition, the widespread use of only one reference gene for normalization of qPCR data has been shown to be inadequate at times often resulting in values that are multiple-fold wrong [3]. It has thus been suggested that using the geometric mean of two or more meticulously selected reference genes results in more accurate comparisons and quantification between study conditions.

In this study, we sought to identify reference genes suitable for determination of gene expression in guinea pig cervical tissues and cells. We also examined the impact of reference gene choice on the measured changes in expression of a gene that has been shown to be expressed and regulated in cervix at term pregnancy in women [4], i.e., *PTGS2*, the gene for cyclooxygenase 2. This gene product increases the levels of bioactive prostaglandins which are known to be of importance in cervical ripening. The temporal relationship between cervical ripening and increase in cervical prostaglandins is not known. In mice, rats, as well as other species that depend on progesterone withdrawal prior to parturition, a combination of loss of progesterone systemically [5] and locally [6] leads to increased prostaglandin biosynthesis in progesterone-responsive target tissues. In primates and guinea pigs, however, progesterone levels do not decline systemically prior to parturition. Studies of events involved in cervical ripening in humans are complicated by difficulties in obtaining cervical tissue from second and third trimesters of pregnancy. Indeed, most studies to date have been carried out on cervical biopsies taken either in connection with early termination of pregnancy [7,8], at the time of vaginal delivery [7,9-11], or Cesarean section at term [10,12,13], usually after cervical ripening. Thus, to study this process both in vivo and in vitro, we chose to use the pregnant guinea pig as a model system to dissect changes in gene expression in hormonal settings that mimic those of humans. Despite its historical popularity in research [14], few studies have been published in which guinea pig gene expression levels have been determined using qPCR. Further, to our knowledge, none have been conducted in cervical tissues or cells.

Several aspects of qPCR studies in the guinea pig cervix are worthy of consideration. First, the cervix is a complex tissue composed of several cell types. Specifically, the stromal compartment is comprised of highly specialized fibroblasts that orchestrate changes in the extracellular matrix and the biomechanical properties of the cervix [15,16], but myofibroblasts and smooth muscle cells are also present. Endocervical epithelial cells line the cervical canal and are known to alter gene expression during cervical ripening and labor [4]. Finally, a number of different immune cells infiltrate and are activated within the cervix and are believed to contribute to matrix remodeling and function of the cervix during pregnancy and parturition [17]. Hence, to quantify changes in gene expression of the cervix as an organ, reference genes must be applicable to many cell types, all of which differ in transcriptional activity.

Here, we used Excel-based applets (geNorm and BestKeeper) to determine the most stable reference genes among 15 candidates for use in qPCR studies of gene expression levels in guinea pig cervical tissues and cell cultures. More specifically, cervical stroma and epithelial tissues from guinea pigs under various hormonal conditions (e.g., immature, mature nonpregnant and pregnant) were collected and analyzed. Likewise, gene expression levels were quantified in primary cervical stromal and epithelial cells in culture treated with or without estradiol and/or progesterone.

## Results

### Primer validation

**Figure 1:**
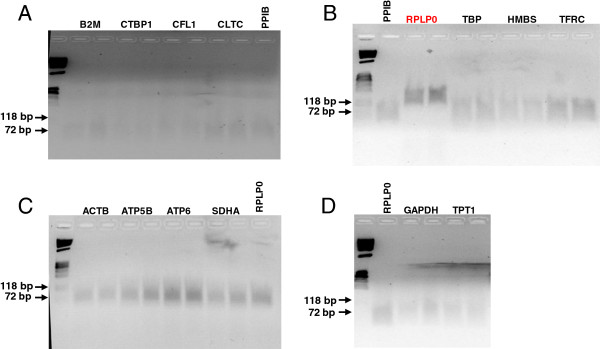
**Primer validation.** Each qPCR reaction mix was loaded in duplicate on 2% Agarose-1000 gels. First RPLP0 primer pair tested (gel **B**) resulted in an amplification product of ~130 bp (should be 61 bp). New primers were designed, and the resulting qPCR reaction was found to be of the correct size, 64 bp (gel **C** and **D**).

The quality of each qPCR primer pair was initially examined using 5-fold dilution series of guinea pig liver cDNA, and acceptable primer combinations had amplification efficiencies greater than 95%, and correlation coefficients ≥ 0.996 (Table 1). Melt-curve analyses revealed a single peak and no amplification in no template controls. Further, examination of qPCR reaction products using 2% agarose-1000 gel electrophoresis revealed a single band of expected size (Figure 1), and gene-specific amplification was confirmed by sequencing the qPCR products.

**Table 1:** Guinea pig reference gene primer validation using liver cDNA

**Gene**	**Slope**	**Amplification efficiency (%)**	**Correlation coefficient**
***ACTB***	−3.4077	96.5	0.9996
***ATP5B***	−3.3395	99.3	0.9997
***ATP6***	−3.4453	95.1	0.9996
***B2M***	−3.2484	103.2	0.9975
***CFL1***	−3.2969	101.1	0.9983
***CLTC***	−3.3034	100.8	0.9973
***CTBP1***	−3.2946	101.2	0.9998
***GAPDH***	−3.3797	97.6	0.9997
***HMBS***	−3.2421	103.4	0.9962
***PPIB***	−3.2028	105.2	0.9981
***RPLP0***	−3.3246	99.9	0.9984
***SDHA***	−3.3581	98.5	0.9999
***TBP***	−3.2961	101.1	0.9979
***TFRC***	−3.3674	98.1	0.9993
***TPT1***	−3.2627	102.5	0.9980

### Expression stability according to geNorm and BestKeeper

Each DNase I-treated RNA sample was analyzed twice, and each qPCR was performed in triplicate. The intra-assay coefficient of variation (CV) ranged from 0.10-0.33 for cervical tissue samples, and from 0.10-0.39 for primary cervical cells. The inter-assay CV for tissues ranged from 0.54-3.06 and from 0.60-2.56 for primary cells. Expression level of the candidate reference genes in each tissue was examined before the geNorm or BestKeeper analyses were performed. Any reference gene for which the Cq was above 30 among in a category of samples was excluded from the study. Expression of *TBP* was found to be too low in all tissues and cell types. *HMBS* expression level was too low in stromal tissues and stromal cells, and *TFRC* expression was also too low in stromal tissues. The remaining candidates were included in the geNorm and Best Keeper analyses to determine expression stability.

Of the remaining candidate genes examined with geNorm, all exhibited expression stability (M) values of less than 1.0 (lower M value indicates a more stable gene) regardless of tissue or cell type (Tables 2 and 3). This is well below the arbitrary cut-off value of 1.5 for acceptable expression stability. Ranking of expression stability as determined by geNorm analysis in cervical guinea pig tissues is shown in Table 2. The two most stable genes were not further ranked by geNorm and are thereby listed together. *CFL1* and *CLTC* were found to be the most stable genes in samples from both stroma and epithelium (i.e., M values of 0.4-0.9 in cervical stroma and 0.3-0.7 in cervical epithelium). BestKeeper scores the reference genes based on a repeated pairwise correlation analysis, and the more stable genes have a Pearson coefficient of correlation (R) that equals to or is close to 1 (Tables 2 and 3). According to this method, the most stable genes in tissues were identical as those identified using geNorm (Table 2). Specifically, *CFL1* and *CLTC* exhibited correlation coefficients ranging from 0.985-0.995. Overall, most genes displayed more stable expression in cultured cells relative to cervical tissues. Stability predictions for other genes were not as concordant between geNorm and BestKeeper.

**Table 2:** Stability of various potential reference genes in guinea pig cervical tissues

	**Stroma enriched**		**Epithelium enriched**	
	**geNorm**	**BestKeeper**	**geNorm**	**BestKeeper**
Most stable	***CFL1/CLTC*** (0.42)	***CLTC*** (0.991)	***CFL1/CLTC*** (0.29)	***CLTC*** (0.995)
	***ACTB*** (0.48)	***ATP5B/CFL1*** (0.985)	*PPIB* (0.34)	***CFL1*** (0.991)
	*ATP5B* (0.51)	*B2M* (0.983)	*SDHA* (0.38)	*SDHA* (0.983)
	*GAPDH* (0.54)	*ACTB* (0.978)	*GAPDH* (0.41)	*TPT1* (0.979)
	*PPIB* (0.57)	*TPT1* (0.977)	*ATP5B* (0.44)	*B2M* (0.970)
	*SDHA* (0.62)	*SDHA* (0.975)	*ACTB* (0.47)	*TFRC* (0.966)
	*ATP6* (0.70)	*RPLP0* (0.967)	*HMBS* (0.51)	*PPIB* (0.965)
	*RPLP0* (0.75)	*ATP6* (0.955)	*TFRC* (0.55)	*ACTB* (0.958)
	*B2M* (0.78)	*CTBP1* (0.954)	*ATP6* (0.57)	*ATP5B* (0.954)
	*TPT1* (0.83)	*GAPDH* (0.952)	*B2M* (0.61)	*HMBS* (0.950)
	*CTBP1* (0.89)	*PPIB* (0.937)	*RPLP0* (0.65)	*ATP6* (0.920)
			*CTBP1* (0.68)	*GAPDH* (0.92)
			*TPT1* (0.72)	*CTBP1* (0.895)
Least stable				*RPLP0* (0.892)
Excluded	*HMBS*		*TBP*	
	*TBP*			
	*TFRC*			

Genes listed with the most stable as determined by geNorm or BestKeeper on top. Genes excluded due to low expression levels in the respective tissue are listed at the bottom. The numbers in brackets represent geNorm M values and BestKeeper R values, respectively.

**Table 3:** Stability of reference gene candidates in guinea pig cervical primary cell cultures

	**Stromal cells**		**Epithelial cells**	
	**geNorm**	**BestKeeper**	**geNorm**	**BestKeeper**
Most stable	***ACTB/PPIB*** (0.09)	***PPIB*** (0.999)	***CLTC/PPIB*** (0.10)	***CLTC*** (0.983)
	*SDHA* (0.12)	***ACTB*** (0.996)	***ATP5B*** (0.11)	***PPIB*** (0.981)
	*B2M* (0.15)	*SDHA* (0.995)	*CTBP1* (0.12)	*ATP5B* (0.975)
	*CLTC* (0.18)	*B2M* (0.984)	*B2M* (0.12)	*CFL1* (0.957)
	*RPLP0* (0.20)	*CLTC* (0.977)	*SDHA* (0.13)	*CTBP1* (0.946)
	*CTBP1* (0.21)	*GAPDH* (0.974)	*HMBS* (0.14)	*ATP6* (0.945
	*GAPDH* (0.23)	*RPLP0* (0.969)	*RPLP0* (0.15)	*TFRC* (0.936)
	*CFL1* (0.24)	*CFL1* (0.958)	*ATP6* (0.16)	*B2M* (0.932)
	*TPT1* (0.25)	*CTBP1* (0.957)	*TPT1* (0.18)	*ACTB* (0.932)
	*TFRC* (0.28)	*TFRC* (0.928)	*GAPDH* (0.20)	*GAPDH* (0.922)
	*ATP5B* (0.30)	*TPT1* (0.919)	*CFL1* (0.22)	*SDHA* (0.867)
	*ATP6* (0.33)	*ATP5B* (0.908)	*ACTB* (0.24)	*HMBS* (0.842)
		*ATP6* (0.857)	*TFRC* (0.26)	*RPLP0* (0.838)
Least stable				*TPT1* (0.602)
Excluded	HMBS		TBP	
	TBP			

Genes listed with the most stable as determined by geNorm or BestKeeper on top. Genes excluded due to low expression levels in the respective tissue are listed at the bottom. The numbers in brackets represent geNorm M values and BestKeeper R values, respectively.

M values (determined with geNorm) were lower in cultured primary cells relative to tissue samples, ranging from 0.1-0.35 in stroma cells and 0.1-0.25 in epithelial cells, suggesting that all tested gene candidates are expressed at rather stable levels in cultured cells. *ACTB* and *PPIB* were ranked as the two most stable genes in primary stroma cells whereas *CLTC* and *PPIB* were found to be the most stable in primary epithelial cells (Table 3). BestKeeper analysis also revealed the same two genes as top ranked (Table 3), with R values ranging from 0.981-0.999.

### Pairwise variation to determine optimal number of reference genes

**Figure 2:**
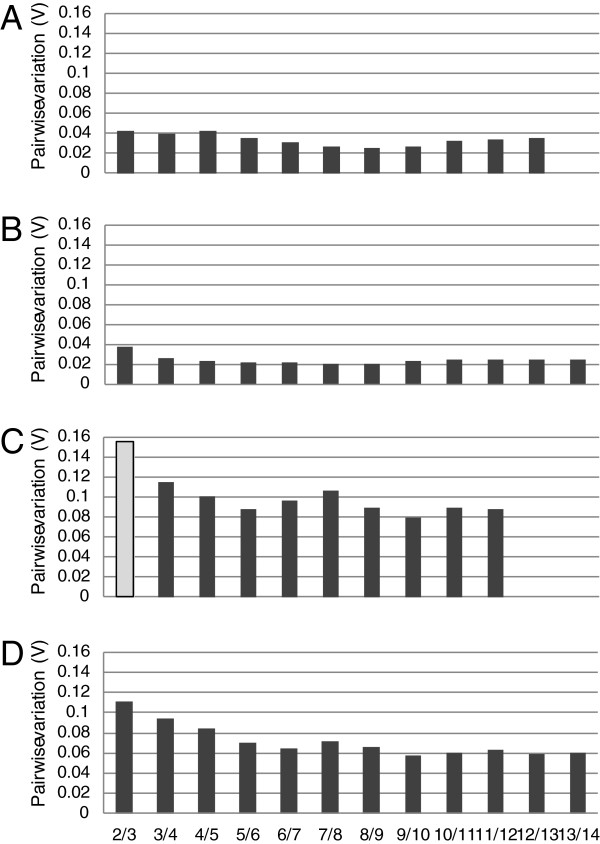
**Pairwise variation of reference gene candidates in cervical cells and tissues as determined by geNorm.** The first bar (2/3) represents the pairwise variation (V) in a 2 vs. 3 reference genes comparison. The second bar (3/4) represents pairwise variations in a 3 vs. 4 reference gene comparison. Variation values were determined using from 2/3 up to 13/14 reference gene comparisons in cultured stromal cells (**A**), cultured epithelial cells (**B**), stromal tissues (**C**), and epithelial tissues (**D**). Pairwise variation values of ≤0.15 were considered sufficient for normalization, values above 0.15 are considered too variable. Note increased V values in tissues compared with cells in culture.

After identification of the most stable genes, the optimal number of reference genes for accurate normalization was determined. Pairwise variation values (V) were determined for each cell type and tissue using geNorm where lower V values correspond to high correlation coefficients. We considered the arbitrary cut-off value for acceptable pairwise variation as 0.15. Using only the two top rated reference genes exhibited values of less than 0.04 in both stroma and epithelial cell. The analysis revealed no substantial benefit of adding more reference genes to the normalization process in these cell types. Specifically, V values were modestly decreased if more reference genes are included (Figure 2A, B). In cervical tissues M values were in general higher and covered a broader range of V values compared with cells in culture (Figure 2C, D). For cervical epithelial tissue (Figure 2D), V values indicated that using two reference genes for normalization still is sufficient whereas three reference genes may be required to achieve good normalization in cervical stroma tissue (Figure 2C).

### Target gene expression levels vary according to choice of reference gene set

**Figure 3:**
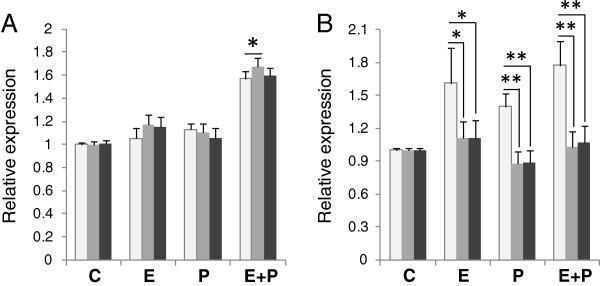
**Effect of normalization options on *PTGS2 *gene expression in cultured cervical cells. ***PTGS2* expression was normalized to either the least stable (white), the top two most stable (gray) or the top three most stable (black) reference genes for each cell type as established with geNorm. Relative levels of *PTGS2* mRNA were determined in (**A**) cervical epithelial cells and (**B**) cervical stromal cells treated with vehicle (C), estradiol (E, 10 nM), progesterone (P, 100 nM), or E+P. Error bars indicate 95% confidence interval; *, p<0.05; **, p<0.01; n=18.

**Figure 4:**
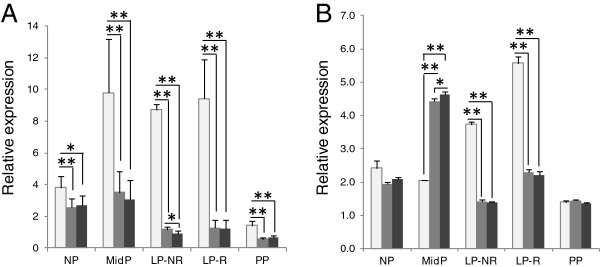
**Effect of normalization options on *PTGS2 *gene expression in cervical tissues.** Example of target gene expression levels in (**A**) cervical epithelial tissues and (**B**) cervical stromal tissues of the target gene when normalized to either the least stable (white), the top two most stable (gray) or the top three most stable (black) reference genes for each tissue type as determined with geNorm. Tissue samples from five guinea pigs representing various hormonal statuses; not pregnant (NP), mid pregnancy (MidP), late pregnancy not ripe (LP-NR), late pregnancy ripe (LP-R), and postpartum (PP). Error bars indicate 95% confidence interval; *, p<0.05; **, p<0.01; n=6.

To determine the effect of different reference gene sets on target gene expression levels, we compared the relative expression of *PTGS2* in cervix-derived tissues and cell samples using different combinations of reference genes. For each sample, qPCR results were normalized to the geometric mean of the top three most stable genes, the top two most stable genes and to the least stable gene (Figures 3, 4). The *PTGS2* expression did not vary significantly in primary cervical epithelial cells regardless of if the top three or only top two reference gene set used. In the comparison between the least stable and the top scoring genes only in one treatment group was there a significant difference in *PTGS2* gene expression (Figure 3A). In primary cervical stroma cells there was no significant difference in *PTGS2* expression when using the top two or three most stable reference genes. However, comparison of any of the most stable gene combinations with the least stable resulted in significant *PTGS2* expression differences (Figure 3B). In cervical tissue samples the effect of using different reference gene sets were more pronounced on *PTGS2* gene expression results, with up to 10-fold difference seen in both some stroma and epithelial samples (not shown). Figure 4 exemplifies the variation in measured *PTGS2* gene expression in five individuals, representing both pregnant and nonpregnant guinea pigs. The difference in *PTGS2* gene expression is significant in almost every sample when compared with the least stable gene, despite the limited sample size. Additionally, the expression level is alternating between higher and lower depending on reference gene set used. Of the examples in Figure 4A if normalized to the least stable gene *PTGS2* expression was greatest in sample GP29, whereas if normalized to expression of *PTGS2* expression was greatest in GP18. Interestingly, levels of the target gene normalized to three recommended reference genes (ACTB/CFL1/CLTC) did not differ appreciably from those normalized to the top two in most stroma samples (Figure 4B).

## Discussion

Gene expression studies in tissue or cell samples depend on usage of appropriate reference genes. Many published qPCR studies normalize target gene expression with the same reference genes that were used for normalization of data obtained with Northern blotting (e.g. *GAPDH, 18S, ACTB* and *HPRT*). These genes have since been found to be regulated in certain situations, for example under the influence of steroid hormones [18-20]. In hormone-responsive tissues, such as the uterine cervix, expression levels of reference genes needs to be unaffected by hormonal change associated with the menstrual/estrous cycle or pregnancy, the effect of the presence of an embryo physical impact on the organ (pressure, stretching,) or growth hormones and prostaglandins released by the growing fetus or placenta. Different methods to find the most stable reference genes have been developed such as geNorm [3], BestKeeper [21], NormFinder [22], Principal Component Analysis [23], and methods based on restricted maximum likelihood with support of descriptive statistics [24]. Kayis et al. [24] compared the ability of these different methods to rank reference gene stability in equine endometrium and found that, with the exception of NormFinder, all methods identified the same genes as the most stable. They also found that all methods gave similar results for the least stable genes in the same tissue. Here we chose to use geNorm and BestKeeper to identify the most stable reference genes of fifteen candidates in tissue and cell samples from the guinea pig cervix.

Although guinea pigs have been used historically and extensively for animal models in the laboratory, the guinea pig genome has not been completely sequenced. When deciding which reference gene candidates to include in this study, we chose to use only genes for which the guinea pig genomic/cDNA sequence was known and we only included mRNA reference genes. We based our exclusion of the commonly used 18S gene not only on the fact that it represents a different class of RNA but it is also present at a very high abundance in samples which makes dilution of the samples necessary conferring less precision and accuracy. Some reference genes included in the study (e.g. *RPLP0*) have been shown to be unregulated by estrogen in human breast cancer cells [25,26]. Similarly, *ACTB* mRNA was reported to be unregulated in human endometrium during the course of the menstrual cycle [27]. Thus, these two genes were of great interest as potential candidates for normalization of qPCR data in the guinea pig cervix. Our results indicate that *ACTB* is indeed a stable gene in guinea pig cervix, but only in stromal tissues and in cultured primary stroma cells. In epithelial tissues and cultured epithelia cells, *ACTB* ranked among the least stable. In addition, *RPLP0* did not rank among the most stable in any of the samples, a finding that exemplifies the importance of evaluating reference for each new sample set and for each experimental set up.

It should be emphasized that cell populations and tissues used in this study are not pure. The collection technique allows us to dissect stroma- and epithelium-enriched tissue. No doubt, each tissue type may be contaminated by epithelial or stromal cells, respectively. Further, the extent of this contamination may vary according to pregnancy or hormonal state. The higher M values in tissue samples relative to those in cultured cervical cells are most likely due to heterogeneous cellular composition of tissues. Nonetheless, results from this study support the notion that even under these very variable conditions it is possible to identify reference genes that are expressed in a stable manner.

## Conclusions

In summary, we used two different methods, geNorm and BestKeeper, to identify the most stable reference genes in guinea pig cervix among fifteen candidates. Both methods identified *CFL1*/*CLTC* as the most stable in cervical tissues, *ACTB*/*PPIB* for cultured stroma cells, and *CLTC/PPIB* for cultured epithelia cells. The minimum number of reference genes to be included in the geometric mean for normalization of qPCR data was found to be two in cervical epithelial tissue and cells, and in cultured stroma cells. In cervical stroma tissue, where the tested reference genes display a higher degree of instability, the mean of three, *ACTB/CFL1/CLTC*, improves the normalization according to geNorm. In our limited sample numbers there was no statistical difference demonstrated between two and three reference genes. Nevertheless, the importance of stable reference genes in quantification of mRNA levels in complex tissues cannot be overstated.

## Methods

### Guinea pig tissue collection

Hartley guinea pigs (Elm Hill Labs, Chelmsford, MA) were kept individually in the Animal Care Facility at UT Southwestern, housed at 21°C with 30-70% humidity under a 12 h light cycle, and fed Teklad Global Guinea Pig Diet 2040 pellets, water, and hay *ad libitum*. All animal procedures were approved by the UT Southwestern Institutional Animal Care and Use Committee. Twenty four female guinea pigs representing sexually immature (n=1), sexually mature non-pregnant (n=4), pregnant (n=15) and postpartum period (n=4) were sacrificed using i.p. Euthasol Euthanasia Solution (390 mg pentobarbital sodium and 50 mg phenytoin sodium/ml) injections according to IACUC approved protocol. The reproductive tract was immediately removed and the cervix excised and further micro dissected into cervical epithelium and stroma. Epithelium was obtained by scraping the lining of the endocervical canal with a scalpel whereas the remaining underlying connective tissue was considered stroma. Cervical and vaginal epithelium covering the external os were discarded. All tissues were snap frozen in liquid nitrogen and stored at −80°C.

### RNA isolation

Cervical tissues (5–50 mg) were thawed and homogenized in RNA STAT-60 (TelTest Inc., Friendswood, TX) using a tissue tearor (Biospec Products, Inc., Bartlesville, OK). Thereafter, total RNA was extracted according to the manufacturer’s protocol, using chloroform (C2432, Sigma, St. Louis, MO), isopropanol (I9516, Sigma, St. Louis, MO), and ethyl alcohol (E190, Pharmaco-Aaper, Brookfield, CT). Each RNA sample (~10 μg) was treated for 30 min @ 37°C with 2 U DNase I in 50 μl reactions (DNA-free, part no. AM1906, Ambion) for removal of contaminating genomic DNA, and subsequently stored at −80°C. Total RNA was isolated from cells in culture using an RNAqueous®-4PCR kit (cat. No. AM1914, Ambion, Austin, TX), and subsequently stored at −80°C.

### Guinea pig cervical primary cell culture

Cervical tissues were obtained from a mature nonpregnant guinea pig. After mincing in 2–4 mm pieces, fresh tissues were incubated for 1 h at 37°C in solution containing collagenase B (1 mg/ml) (cat no. 11088823103, Roche, Indianapolis, IN) and DNase I (0.1 mg/ml) (cat no. 10104159001, Roche, Indianapolis, IN). The mixture was then strained through a 70 μm mesh (cat no. 352350, BD Biosciences, Bedford, MA) to achieve separation between stromal (flow through) and epithelial (retained) cells. The respective cells were collected, dispersed in DMEM without phenol red (cat no. 11054, Invitrogen, Carlsbad, CA) buffered with 10 mM HEPES (cat no. 15630, Invitrogen, Carlsbad, CA), and supplemented with 2 mM L-Glutamine (cat no. 25030, Invitrogen, Carlsbad, CA), 10% by volume fetal bovine serum (FBS) (cat no. S11150, Atlanta Biologicals, Lawrenceville, GA) and penicillin G (10 U/ml)-streptomycin sulfate (10 μg/ml)-amphotericin B (25 ng/ml) mix (cat no. 9350, Irvine Scientific, Santa Ana, CA). Cells were plated at 5 × 10^5^ cells/cm^2^ and culture medium was changed every other day until near confluency (2–4 day). Preconfluent cells in passages 1–3 were used for experiments. Cells were serum-deprived for 3 day before treatment with 17β-estradiol (10 nM), progesterone (1 μM), a combination of 17β-estradiol and progesterone, or vehicle (ethanol) for 24 h.

### cDNA synthesis

Nucleic acid quantification and purity assessment were conducted spectrophotometrically using a SmartSpec™3000 (Bio-Rad, Hercules, CA), with expected 260/280 ratios above 1.8. A High Capacity cDNA Reverse Transcription Kit (part no. 4368813, Applied Biosystem, Carlsbad, CA) based on random primer priming with MultiScribe™ Reverse Transcriptase was used for cDNA synthesis from 2 μg total RNA in 20 μl reaction volumes, with the following reaction conditions; 25°C for 10 min, 37°C for 2 hrs, 85°C for 5 min, and 4°C for 1 min. The final product was diluted to correspond to 20 ng initial RNA input/μl, and was stored at −20°C.

### Selection of reference and target genes, and primer design

Potential reference genes were chosen among those used in various human tissues or cell types [3,23,28,29] for which the corresponding guinea pig nucleotide sequence was available. When possible, primer pairs were designed to span an intron. Two primer sets have been used previously as guinea pig reference genes in qPCR assays, *GAPDH*[30] and *ACTB*[31]*.* For target gene we chose cyclooxygenase-2, *PTGS2*, an enzyme involved in prostaglandin synthesis that has been shown to be expressed in human cervix [4]. Primer sequences are shown in Table 4. All primers were synthesized commercially (Integrated DNA Technology, Coralville, IA).

**Table 4:** Guinea pig reference and target gene information

**Gene symbol**	**Name**	**Primer sequences 5’ to 3’**	**Exons**	**Amplicon size (bp)**	**Function**	**Ensembl or GenBank accession no.**
		**Sense and antisense**				
*ACTB*	β-Actin	tgcgttacaccctttcttgaca	5	73	Cytoskeletal protein	From ref. [31]
		acaaagccatgccaatctcat				
*ATP5B*	ATP synthase, F1 complex β subunit	gatcaatttaaaagatgctacctcgaa	5 and 6	68	ATP synthesis	DQ403103
		caccaggcggttcattcatt				
*ATP6*	Adenosine triphosphatase 6	cccactatgagcagcaactgtaa	1	67	ATP metabolism	NC_000884
		gaagtgggctagggatgcttt				
*B2M*	β2-Microglobulin	tggtgcatgctgcctttaca	2	64	Cell surface molecule component	NM_001172856
		gtgatgtgtgaaactctgcaagaa				
*CFL1*	Cofilin 1	ttccaaggatgccatcaaaaa	3 and 4	65	Actin modification	ENSCPOT00000008138
		cgtagcaatttgcctgtaattcg				
*CLTC*	Clathrin	caattcgttttcaggagcatctc	1 and 2	64	Coated vesicle formation protein	ENSCPOT00000005567
		aagccaatgtttgctgggtta				
*CTBP1*	C-terminal binding protein 1	tctcatcaacgacttcactgtcaa	4 and 5	61	Transcriptional repressor phosphoprotein	ENSCPOT00000019529
		ggccgtgttcaccaggaa				
*GAPDH*	Glyceraldehyde-3-phosphate dehydrogenase	tcagagggctccctcaaag	2	70	Glycolytic enzyme	From ref. [30]
		cgctgttgaagtcacaggac				
*HMBS*	Hydroxymethyl-bilane synthase	cctgggttggcagaacaga	9 and 10	66	Heme biosynthesis	ENSCPOT00000006534
		tggcccacagcatacatacag				
*PPIB*	Cyclophilin B	gggcctaaagtcaccgtcaa	1 and 2	63	Protein folding catalyst	ENSCPOT00000000258
		ccggcccacatcttcatct				
*RPLP0*	Ribosomal protein, large, P0	atgctgctggccaataaggt	3 and 4	64	Protein synthesis	ENSCPOT00000019555
		tgacttcacatggtgcaatgg				
*SDHA*	Succinate dehydrogenase, subunit A	gatgccatccattacatgacaga	4 and 5	67	Citric acid cycle enzyme	DQ402978
		gcatgccataattttctagctcaa				
*TBP*	TATA-binding protein	acttgacctaaagacaattgcacttc	5 and 6	65	Transcription factor	ENSCPOT00000001200
		cagcaaaccgcttgggatta				
*TFRC*	Transferrin receptor	gaccttccagtcttcggtcatg	8 and 9	68	Iron transport	S81327
		aaagaagggaacccaggtgtataa				
*TPT1*	Tumor protein, translationally controlled 1	ccttgctaatttcaaaaactatcagttc	4 and 5	67		EU330893
		agcaaccatgccatctggat				
*PTGS2*	Cyclooxygenase-2	ctgcgcaatgcaatcatga	3 and 4	67	Prostaglandin synthesis	Y07896
		agttggtggactgtcgatcaga				

### Quantitative real-time PCR (qPCR)

All qPCR was performed using 50% of iTaq SYBR green SUPERMIX with ROX (cat. no. 172–5851, Bio-Rad, Hercules, CA), 900 nM of each primer and 30 ng cDNA (assuming a 1:1 reversed transcription reaction efficiency) in 19 μl reactions in MicroAmp® Optical 384-Well Reaction Plates (part no. 4309849, Applied Biosystems, Carlsbad, CA) on a 7900HT Fast Real-Time PCR System (Applied Biosystems, Carlsbad, CA). The cycling program was: stage A 50°C for 2 min; stage B (denaturation) 95°C for 10 min; stage C (cycling) 95°C for 15 sec and then 60°C for 1 min repeated 40 times, and stage D (dissociation) 95°C for 15 sec then 60°C for 15 sec and a final 95°C for 15 sec. All samples were run in triplicate and each primer pair was validated using a 7 point 5-fold dilution series of guinea pig liver cDNA (50–0.0032 ng per reaction) and no template controls.

### Analyses of qPCR results using geNorm and BestKeeper

Each RNA sample was analyzed twice from the reversed transcriptase step and on, and the threshold cycle (Cq) value for each sample was determined for all primer pairs. The mean Cq of triplicates for each sample run was then used for input in geNorm v3.5 and BestKeeper v1 applets, and the respective stability values were automatically calculated as described [3,21].

## Abbreviations

qPCR: Quantitative real-time PCR; NTC: No template control; Cq: Threshold cycle; ACTB: β-actin; ATP5B: ATP synthase F1 complex β subunit; ATP6: ATPase 6; B2M: β2-microglobulin; CFL1: Cofilin 1; CLTC: Clathrin; PTGS2: Cyclooxygenase-2; CTBP1: C-terminal binding protein 1; GAPDH: Glyceraldehyde-3-phosphate dehydrogenase; HMBS: Hydroxymethyl-bilane synthase; PPIB: Cyclophilin B; RPLP0: Ribosomal protein large P0; SDHA: Succinate dehydrogenase subunit A; TBP: TATA-binding protein; TFRC: Transferrin receptor; TPT1: Tumor protein translationally controlled 1; FBS: Fetal bovine serum.

## Competing interests

All authors declare that they have no competing interest.

## Authors’ contributions

AL conceived the study and participated in its design, participated in tissue retrieval and cell culture, performed all qPCRs, and drafted the manuscript. DM participated in cell culture and performed the computer and statistical analyses. RAW participated in the design of the study, participated in tissue retrieval, and helped draft the manuscript. All authors read and approved the final manuscript.
